# Modification of sodium aescinate into a safer, more stable and effective water-soluble drug by liposome-encapsulation: an *in vitro* and *in vivo* study

**DOI:** 10.1080/10717544.2022.2058114

**Published:** 2022-04-05

**Authors:** Sifan Huang, Xinyu Wang, Mengmeng Liu, Zhizhe Lin, Wenqian Gu, Haili Zhao, Yanqiu Zhang, Baoyue Ding, Jiyong Liu, Xin Wu, Wei Fan, Jianming Chen

**Affiliations:** a Department of Pharmacy, Fujian University of Traditional Chinese Medicine, Fuzhou, China; b Shanghai Wei Er Lab, Shanghai, China; c Department of Pharmacy, Jiaxing University, Jiaxing; d Department of Pharmacy, Fudan University Shanghai Cancer Center, Shanghai, China; e Seventh People's Hospital of Shanghai University of Traditional Chinese Medicine, Shanghai, PR China

**Keywords:** Sodium aescinate, liposome, stability, safety, efficiency

## Abstract

Sodium aescinate (SA) is often used for intravenous (IV) injection owing to its anti-inflammatory, anti-exudative, increasing venous tension, improving blood circulation and reducing swelling activities. However, the clinical application of SA is limited by strong irritation, short half-life and low bioavailability. To overcome these defects, we intended to modify SA by encapsualing it with liposomes . SA was mixed with a proper amount of phospholipid and lyophilized to prepare the liposome of sodium aescinate for injection (SA-Lip-I). Its physical properties, cumulative release and dilution stability were evaluated in vitro. Its pharmacodynamic characteristics were evaluated. Safety of SA-Lip-I was evaluated in terms of hemolysis, IV irritation and acute toxicity. The mean particle size of SA-Lip-I was 117.33±0.95 nm, polydispersity index (PDI) was 0.140±0.017, Zeta potential was -30.34±0.23 mv, The cumulative release of SA-Lip at 12 h was more than 80%, which met the release requirements of nanoparticles. SA-Lip-I was well stable in the four mediators and met the clinical medication requirements. In addition, SA-Lip-I had better efficacy than the SA-I and has a significant difference. Furthermore, SA-Lip-I did not induce hemolysis at 37°C, and produced by far milder venous irritation as compared with SA-I. In addition, LD50 of SA-Lip-I was 2.12 fold that of the commercial SA-I, with no obvious side effects.The modified SA-Lip-I is a promising preparation which can reduce the irritation and toxic side effects, improve the treatment effect to a certain extent, but greatly alleviate pain of the patient during treatment, achieving the optimal curative effect.

## 1. Introduction

SA is a triterpenoid saponin sodium salt extracted from dried mature fruits of Chinese Buckeye Seed, with anti-inflammatory, anti-osmotic and anti-swelling effects (Costantini, 1999; Song et al., 2016). Clinically, it is mainly used to treat edema and hematoma in various parts of the human body, acute and chronic tissue injuries, bone fracture, trauma, brain dysfunction due to various reasons, and local blood circulation disorder caused by various vascular diseases (Amould et al., 1996; Gallelli, 2019). Clinical experiments have demonstrated that SA can be used as a stable, effective and reliable anti-inflammatory and anti-swelling drug.

The liniment, gel, tablet and injection forms of SA have been widely used in clinical practice. Among them, SA liniment and gel for external use show good therapeutic effects on ecchymosis, sprain, crush injury and other local surface injuries. However, external preparations have certain limitations. for instance, they cannot provide in-depth treatment for various vascular diseases caused by local blood circulation disorders. In addition, they cannot be used on the damaged skin and mucous membrane (Lang, 1974; 1977). SA tablet is easy to be administered orally, but its poor absorption and low bioavailability limit its use (Lang & Mennicke, 1972; Wu et al., 2012). SA-I has the advantages of rapid action and a long maintenance time of the biological effect. It can effectively treat and prevent postoperative edema and alleviate pain of the patient (Wang et al., 2016; Liyou et al., 2018). However, IV (IV) injection of SA-I can cause pain in the injection site because of the strong irritation of septate sodium to blood vessels, and prolonged medication may cause phlebitis. Meticulous observation is therefore necessary during infusion to prevent fluid leakage to surrounding tissues, causing red swelling. In addition, it is not convenient to prepare SA-I because bubbles are likely to appear during preparation (Feng et al., 2011; Shi et al., 2014; Xie et al., 2014).

At present, liposomes, as a new nanoscale drug carrier, have been widely concerned. Liposomes can wrap around lipid-soluble and water-soluble drugs. However, because the outer aqueous phase of liposomes is greater than the internal aqueous phase, water-soluble drugs tend to accumulate in the outer aqueous phase, resulting in drug leakage, generally low encapsulation efficiency (EE) and poor stability. Therefore, the clinical application of the water-soluble drug liposomes is limited.

Based on the disadvantages of the above liposome-coated water-soluble drugs, the currently listed water-soluble drugs such as ONIVYDE (Drummond et al., 2006; Syrigos, 2016), VYXEOS (Dicko et al., 2010), EXPAREL and DEPODUR improve the above problems by preparing the active loading method and Re-emulsion method. However, the liposomes prepared by the active drug loading method and Re-emulsion method have poor stability, prone to flocculation, uneven particle size, complex preparation process, long operation time, and difficult industrial production.^^®^^®^®®^

In this experiment, the ethanol injection method was used to prepare sodium aescinate liposomes by adjusting the ratio of phospholipids to cholesterol, the content of organic solvents and the ratio of drug-to-lipid, which not only has a high EE%, but also has good stability. Reduced its direct contact with the vascular wall, thus reducing its IV irritation, improve hemolysis, and more importantly, can improve efficacy and improve patient compliance. The particle size, PDI index and Zeta potential were characterized and the release and physical stability in vitro were evaluated. We compared the pharmacodynamic characteristics of SA-Lip-I and SA-I in paraxylene-induced ear swelling in mice, cotton pellet granuloma in rats and carrageenan-Induced paw edema in rats, and compared the differences between the two dosage forms at the level of inflammatory factors. The safety of SA-Lip-I was evaluated by hemolysis, IV irritation and acute toxicity.

## 2. Materials and methods

### 2.1. Materials

Materials used in this study included SA (Xi'an Yanhao Biotechnology Co., Ltd., Xi’an, China), sodium aescinate for injection (Hunan Yige Pharmaceutical Co., Ltd., Hunan, China), EPCS (Germany Lipoid GmbH), DSPE-PEG200 (Shanghai Libold Biotechnology Co., Ltd., Shanghai, China), cholesterol and xylene (Shanghai Titan Technology Biotechnology Co., Ltd., Shanghai, China), Carrageenan (Shanghai Yuanye Biotechnology Co., Ltd., Shanghai, China); Elisa kits for TNF-α, IL-Iβ and PEG2 (BioChannel Biological Technology Co., Ltd., Nanjing, China), and 0.9% normal saline (NS) and 5% glucose injection (Sichuan Collen Pharmaceutical Co., Ltd., Sichuan, China). All other solvents were of chromatographic grade and other chemicals were of analytical grade.

### 2.2. Animals

ICR mice (18–22 g), SD rats (160–220 g) and New Zealand big-ear rabbits (2 kg) purchased from Fujian University of Traditional Chinese Medicine (Fuzhou, China) were kept at 25 ± 2°Cwith a relative humidity of 47.5 ± 2.5% in our laboratory. All animal experiments were conducted in accordance with the ethical guidelines issued by the said Fujian University of Traditional Chinese Medicine on the investigation of experimental animals. Animal care and treatment were strictly in line with the Laboratory Animal Care and Use Guide.

### 2.3. Sample analysis by high performance liquid chromatography (HPLC)

HPLC analysis was performed using Waters e2695II HPLC system (Waters, Billerica, MA). Briefly, samples were separated using an Agilent EclipsePlus C18 column (250 × 4.6 mm, 5 μm). The mobile phase was a mixture of acetonitrile and 0.55% phosphate saline solution (37:63, v/v) at a flow rate of 1.0 mL/min. The absorbance was measured at 220 nm.

### 2.4. Preparation and characterization of sodium aescinate liposome

The preparation steps of SA-Lip-I were as follows: the prescription amount of EPCS, cholesterol and DSPE-PEG2000 was weighed out accurately and dissolved in anhydrous ethanol. The organic phase was dissolved by heating at 55 °C. An appropriate amount of SA was dissolved in injection water by adjusting the pH value and keeping heating at 55 °C to obtain the aqueous phase. By slow addition of the organic phase under magnetic stirring, the crude product of sodium aescinate liposome (SA-Lip) was obtained, under the same extrusion pressure and temperature, the liposome was extruded through the 100 nm polycarbonate membrane to obtain SA-Lip. The freeze-dried product of SA-Lip-I was obtained after ultrafiltration to remove the organic solvent and 12% sucrose solution over an 0.22 μm microporous membrane as a lyophilization protective agent, freeze-dried and sealed to obtain freeze-dried the product SA-Lip-I.

### 2.5. Characterization of SA-Lip

The particle size, PDI, and potential of SA-Lip-I were determined by using the Malvern particle size analyzer (Malvern, USA). In addition, the morphology of liposomes was characterized by transmission electron microscopy (TEM) (Deshmukh et al., 2021). The sample was prepared as follows: a proper amount of SA-Lip-I solution was dropped on the surface of the carbon-sprayed copper mesh and deposited into the copper mesh, and dyed with 2% phosphotungstic acid for 3 min. The morphology of SA-Lip-I was observed under a high-resolution TEM after natural drying.

The EE% of SA-Lip was assessed by ultrafiltration centrifugation by applying an appropriate amount of the sample to a 50 kD ultrafiltration centrifuge tube, and centrifuged by ultrafiltration at 4500 r/min for 10 min to separate the free drug and liposome. The free drugs from the recovery chamber and total drugs were determined by HPLC at 220 nm (Cheng Ying et al., 2021). EE% was calculated using the formula below:

$$EE\%=\frac{C_{\text{total}}-C_{free}}{C_{total}}*100\%$$

where *C*_total_ is the total amount of SA in liposome and *C*_free_ is the amount of free SA in liposome.

### 2.6. The release of SA-Lip-I

The release kinetics of SA was quantitatively detected by drug release test. A portion of (1.5 mL) of SA-Lip and SA-I was added to the pretreatment dialysis bag and fixed to the paddle of the dissolution device. The release middle is PBS (pH 5.5), PBS (pH 7.4) at 37.0.0 ± 0.5 °C and the blade speed at 100 rpm. Sample solution was extracted at certain intervals and the same volume of fresh middle was immediately added. Drug concentrations in the samples were analyzed by the HPLC assay. The drug release curve was calculated by the following formula:

$${\text{E}}_{\text{r}}\left(\%\right)=\left[\frac{C_{n}}{L/V_{2}}+\frac{\frac{\left(C_{n-1}+...+C_{2}+C_{1}\right)}{L/V_{2}}V_{1}}{V_{2}}\right]\times 100\%$$

where *C*_n_ is the sample concentration after removal at each time point; L is the preparation quantity; V_1_ is the the fixed sampling volume at each time point; V_2_ is the dissolution middle volume.

### 2.7. Physical stability study

The stability of SA-Lip reconstitution was evaluated 24 h after reconstructed at 4 °C. The SA-Lip-I was reconstructed with water for injection and stored at 4 °C. The particle size, PDI and EE% of the sample were analyzed at different time points.

SA-Lip-I was diluted with 5% glucose or 0.9% sodium chloride injection to the same concentration as SA-I (1 mg/mL). The diluted sample was observed at a temperature of 25 ± 2 °C. The particle size, PDI and EE% of the sample were analyzed at different time points. The stability of SA-Lip-I diluted with 0.9% saline and 5% glucose was evaluated.

Since SA-Lip-I is an IV injection, we examined its physical stability in serum, simulating the *in vivo* serum system, and indirectly examining SA-Lip-I particle size changes using altered light transmittance. The specific methods are as follows: NS was prepared with 10 and 50% FBS. SA-Lip was mixed with the two volumes of NS at a 4-fold ratio, incubated for 0, 2, 4, 6, 12 and 24 h at 37 °C. Absorbance was determined at 750 nm. The stability was evaluated using the ratio of SA-Lip in 10–50% FBS NS to their absorbance in NS.

### 2.8. *In vivo* pharmacodynamic study

#### 2.8.1. Paraxylene-induced ear swelling in mice

Forty ICR mice were equally randomized into five groups: a blank group, an SA-I group (3.6 mg/kg), and three SA-Lip-I injection dose groups at 1.8 mg/kg, 3.6 mg/kg, and 7.2 mg/kg, all for three consecutive days. Thirty minutes after the last drug administration, the right ear of each rat was coated with 30 μL xylene, and the left ear without xylene coating was used as the control. Thirty minutes after inflammation induction, the mice were killed. Both left and right ears were cut off along the auricle baseline. An ear flap was punched off from each ear with an 8-mm perforator at the same location, and weighed with an electronic balance. The weight difference (mg) between the left and right ears was used as the swelling rate, based on which the swelling inhibition rate was calculated (Zhao et al., 2018).

#### 2.8.2. Cotton pellet granuloma in rats

Thirty-five SD rats were equally randomized into five groups: a blank group, an SA-I group (1.8 mg/kg), and three SA-Lip-I injection dose (0.9 mg/kg, 1.8 mg/kg, and 3.6 mg/kg) groups. The drug was administered for 7 consecutive days. One day before drug administration a sterile cotton ball (35 mg) was implanted in each rat groin the day before the first drug administration. 24 h after the last drug administration, the cotton ball was removed together with the surrounding connective tissue, and after removing the fat tissue, dried in 60 °C drying oven for 12 h and weighed. The weight of the granulation was obtained by subtracting the weight of the cotton ball (Eisenburger et al., 1976; Zhao et al., 2018).

#### 2.8.3. Carrageenan-induced paw edema in rats

Thirty-five SD rats were equally randomized into five groups: a blank group, a SA-I group (1.8 mg/kg), and three SA-Lip-I injection dose (0.9 mg/kg, 1.8 mg/kg and 3.6 mg/kg) groups. The drug was administered for 5 consecutive days. One hour after the last drug administration, 0.1 mL1% carrageenan solution was injected to induce rat foot swelling. The volume of the paw was measured by water quality balance method 1 h before carrageenan injection, and 1 and 4 h after injection to calculated the degree of swelling (Wang et al., 2013; Zhao et al., 2018).

### 2.9. Elisa for PGE2, TNF-α, and IL-1β analyses

Plasma obtained from the rat toe edema model was centrifuged at 15,000 rpm for 10 min to obtain the serum sample. TNF-α, IL-1β, and PGE2 were detected with ELISA kits according to manufacturer's instructions (Zhao et al., 2018).

### 2.10. Safety assessment

#### 2.10.1. IV irritation evaluation

Six rabbits weighing (1.8–2.0 kg) were equally randomized into two groups: a liposome of SA-Lip-I group and a SA-I group. SA-Lip-1 and SA-I were injected IV through the marginal auricular vein of right ear at the dose of 1.3 mg/kg and injection rate of 1 mL/min daily for three consecutive days. At the same time, the left ear was given an equal volume of NS as control. Changes in animal behavior and the injection site were observed with the naked eye during daily administration. The animals were euthanized 24–48 h after the last drug administration. The auricular vein tissue was fixed in 10% paraformaldehyde solution, dehydrated with ethanol gradient, paraffin-embedded, and stained with hematoxylin and ovalbumin. All samples were examined to assess any pathological change using a BX43-DP21 light microscope (Olympus, Tokyo, Japan) (Chen et al., 2017).

#### 2.10.2. Hemolysis test

Hemolysis test was used to evaluate the safety of clinical use of SA-Lip-I. About 20 mL blood was drawn from the rabbit marginal auricular vein. Fibrinogen was removed with a glass rod to obtain the defibrillated blood, which was then mixed thoroughly with 10-fold NS, centrifuged at 2500 rpm for 5 min. after removing the supernatant, the deposited red blood cells (RBCs) were washed with NS for 2–3 times until the supernatant became completely clear. The RBCs thus obtained were diluted with NS by the volume ratio into a 2% RBC suspension by volume.

Both SA-Lip and SA-I were diluted with NS to the clinical concentration of 0.04 mg/mL as specified in the instruction manual for later use. Eight glass tubes containing 2.5 mL 2% RBC suspensions were numerically labeled. Different doses (0.1, 0.2, 0.3, 0.4 and 0.5 mL) of SA-Lip-I were added to test tube 1–5 (T 1–5) respectively. T 6 contained only 2.5 mL 0.9% NS as negative control, T 7 contained 2.5 mL SA-I as a reference control, and T 8 contained 2.5 mL distilled water as positive control. NS was added to each tube to a total volume of 5 mL, mixed thoroughly, incubate at 37 ± 0.5 °C, and whether hemolysis observed at different time points (Chen et al., 2017).

#### 2.10.3. Acute toxicity

Determination of half lethal dose LD_50_ of SA-Lip-I was determined. Briefly, 70 ICR mice were equally randomized at a 1:1 male/female ratio to seven groups according to the adjacent dose ratio of 1:8 (3.958 mg/kg, 4.948 mg/kg, 6.185 mg/kg, 7.731 mg/kg, 9.664 mg/kg, mg/kg, 12.08 mg/kg and 15.01 mg/kg). After animal grouping and dose calculation, the drug was injected via the tail vein as designed. Seven days after drug administration, the number of animal deaths was calculated and recorded. LD_50_ and 95% confidence interval (CI) were calculated with SPSS software (version 21.0) using the weighted linear regression (Bliss) method) (Chen et al., 2017; Gong et al., 2019).

### 2.11. Statistical analysis

Data were statistically analyzed using GraphPad Prism (version 6, San Diego, CA) and SPSS 21 software. All studies were repeated three times and all measurements were carried out in triplicate. Results are reported as the mean ± standard deviation (SD). Differences between experimental groups were considered significant when the *p*-value was less than .05.

## 3. Results

### 3.1. Physical characteristics of SA-Lip

**Figure 1.**
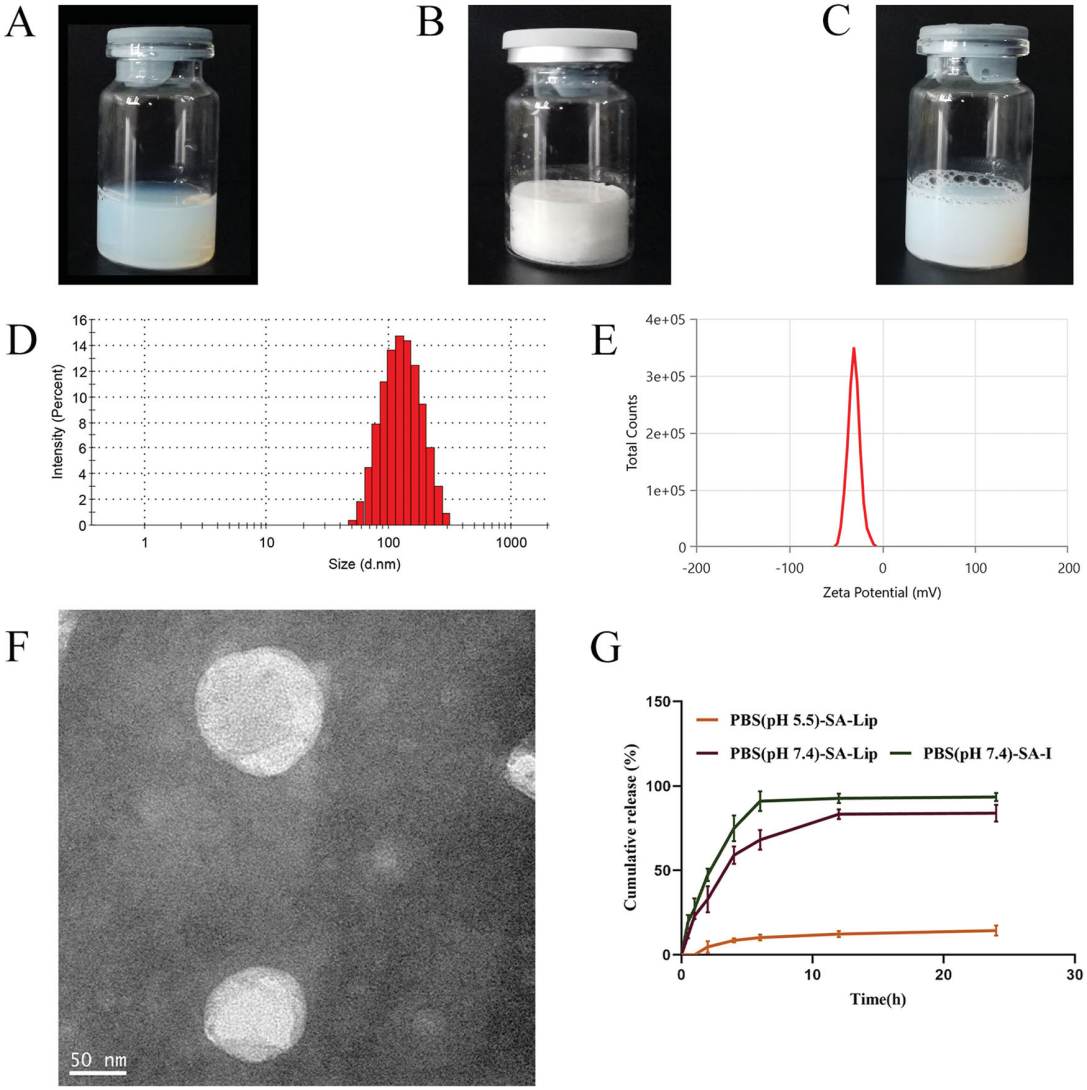
The characteristics of SA-Lip-I. (A) SA-Lip. (B) SA-Lip lyophilized product. (C) SA-Lip reconstituted solution. (D) Particle size distribution of SA-Lip-I. (E) Zeta potential of SA-Lip-I. (F) TEM micrographs of SA-Lip-I. (G) *In vitro* cumulative release of SA-Lip-I in different media (*n* = 3).

The preparation of SA-Lip by ethanol injection, with a simple process and reproducible, and the results showed that SA-Lip had no flocculation or stratification, with uniform dispersion and good fluidity, freeze-dried products was plump with a smooth surface and good fluidity (Figure 1(A–C)). Malven particle size was determined using a particle size analyzer, showing that the size of liposome was 117.33 ± 0.95 nm, PDI was 0.140 ± 0.017 with a single peak (Figure 1(D)), and Zeta potential was −30.34 ± 0.23 mV (Figure 1(E)). TEM imaging showed that the SA-Lip particle was spherical with a narrow particle size distribution, indicating that SA-Lip had good dispersion performance (Figure 1(F)).

As shown in Table 1, the mean EE of SA-Lip was 98.33 ± 0.0012 (%) by ultrafiltration centrifugation, indicating that the result of the method was accurate and the error was small, meeting the requirements of subsequent experiments.

**Table 1.** The encapsulation rate of SA-Lip (*n* = 3).

Production batch	*C*_total_ (mg/mL)	*C*_free_ (mg/mL)	EE (%)	Mean ± SD
20210104	2.49	0.04	98.47	98.33 ± 0.0012
20210105	2.35	0.04	98.17	
20210106	2.45	0.04	98.34	

As shown in Table 2, we compared the EE%, particle size and PDI of liposomes before and after lyophilization of different batches of SA-Lip. The experimental results are shown in the following table: EE% decreased slightly after reconstitution of SA-Lip-I, while particle size and PDI increased slightly, but they were all within a reasonable range. The difference between before and after freeze-drying was small, and the quality of freeze-dried products was good.

**Table 2.** Comparison of SA-Lip-I before and after lyophilization (*n* = 3).

Production batch	Before lyophilization			After lyophilization		
	EE%	Size (nm)	PDI	EE%	Size (nm)	PDI
20210309	99.33	118.23	0.147	95.81	126.40	0.167
20210317	99.43	111.4	0.152	95.58	119.03	0.157
20210322	99.62	117.5	0.141	95.40	129.8	0.164
Mean ± SD	99.46 ± 0.0012	115.71 ± 3.0622	0.147 ± 0.0045	95.60 ± 0.0017	125.08 ± 4.4953	0.163 ± 0.0042

### 3.2. Drug release *in vitro*

The release of SA-Lip and SA-I is shown in Figure 1(G), and the release rate of SA-Lip-I in the PBS5.5 release middle was very slow. It was possible that our SA-Lip was weakly acidic and was relatively stable in a weakly acidic buffer, thus caused it was slow release in PBS5.5. The cumulative release of SA-I aqueous solution could reached over 95.00% after 6 h, while SA-Lip release was slow in the same release middle (PBS7.4) and reached 83.34% at 12 h, with a significant slow-release effect compared with SA-I aqueous solution.

### 3.3. Physical stability test

**Figure 2.**
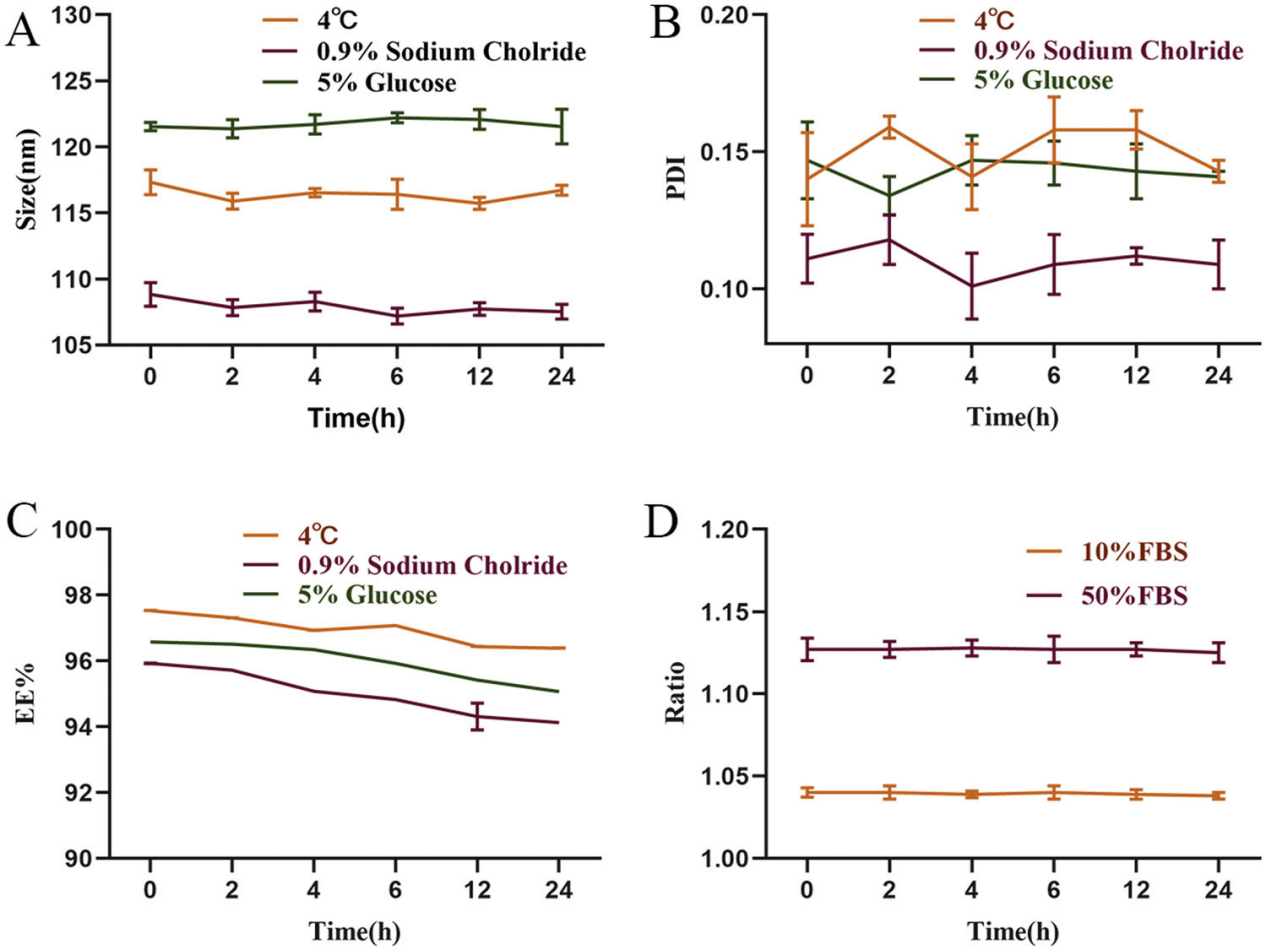
The physical stability of SA-Lip-I. (A) Changes in particle size after SA-Lip reconstitution when diluted with 5% glucose injection and 0.9% sodium chloride injection (*n* = 3). (B) Change in PDI after SA-Lip reconstitution when diluted with 5% glucose injection and 0.9% sodium chloride (*n* = 3). (C) Change in EE% after SA-Lip reconstitution when diluted with 5% glucose injection and 0.9% sodium chloride injection (*n* = 3). (D) Stability of SA-Lip-I in different concentrations of FBS (*n* = 3).

The physical stability of SA-Lip-I was evaluated, and the results are shown in Figure 2(A–D). We evaluated the stability after SA-Lip-I reconstruction within 24 h at 4 °C (Figure 2(A–C)). the mean size of SA-Lip was 116.44 nm, the mean PDI was 0.150, and the mean EE was 96.95%. The stability of SA-Lip in 0.9% NS and 5% glucose was evaluated by SA-Lip diluted to 1 mg/mL as described in the SA-I instructions (Figure 2(A–C)). The mean size of SA-Lip diluted with 0.9% NS was 107.91 nm within 24 h, the mean PDI was 0.110, and the mean EE was 95.00% at room temperature. The results are shown in Figure 2(A–C). At room temperature, the mean particle size of SA-Lip diluted with glucose within 24 h was 121.73 nm, the mean PDI was 0.143, and the mean EE was 95.98%. No significant change was observed in particle size, PDI and EE within 24 h under the three conditions. In addition, SA-Lip-I remained stable in both 10 and 50% FBS (Figure 2(D)). SA-Lip-I was stable within 24 h under the four conditions and met the requirement for clinical administration.

### 3.4. Pharmacodynamic evaluation

#### 3.4.1. Paraxylene-induced ear swelling in mice

**Figure 3.**
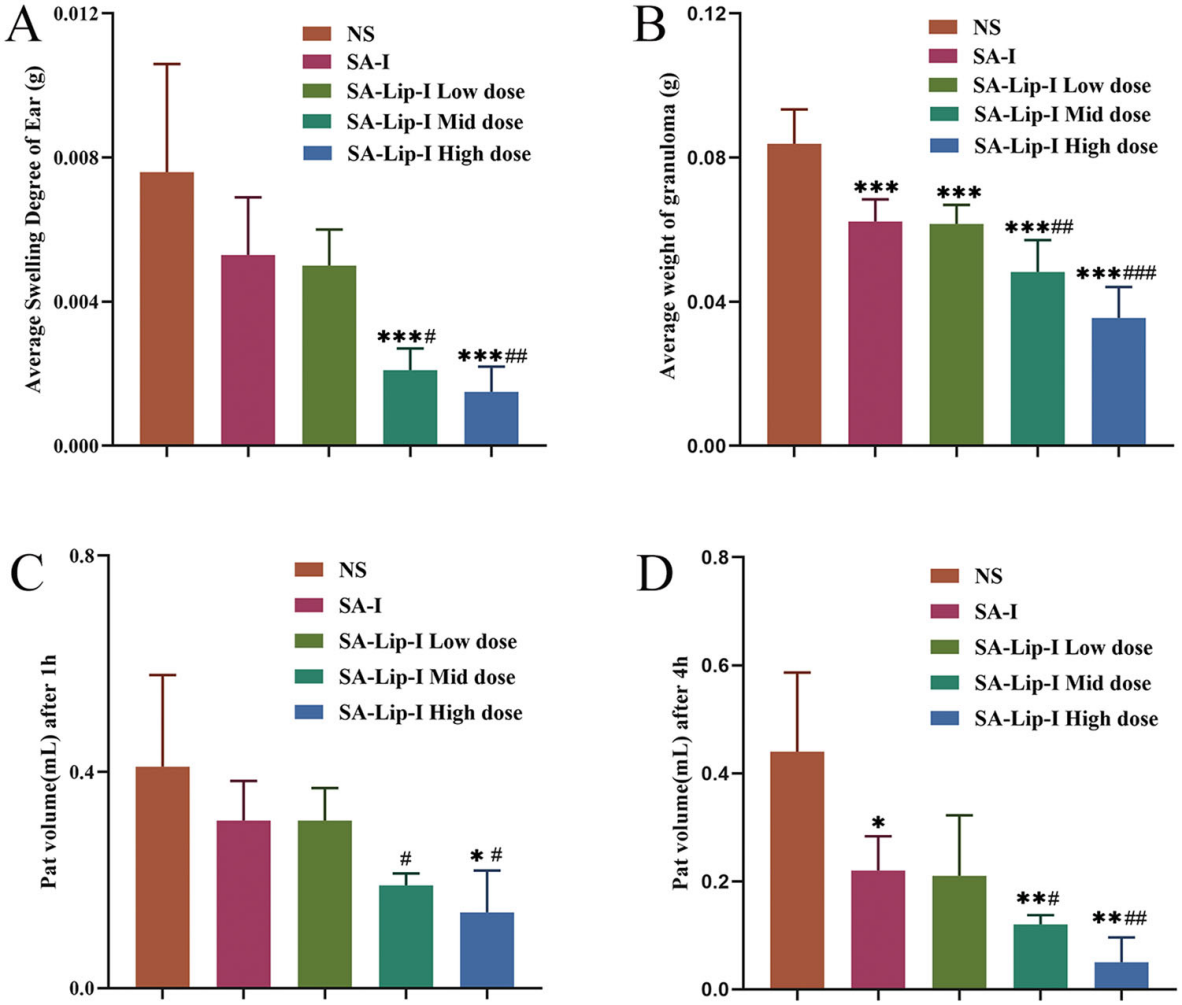
Anti-inflammatory effect of SA-Lip-I *in vivo*. Compared with control group *p < .05, **p < .01, ***p < .001, compared with the SA-I group *p* < .05, *p* < .01, *p* < .001. (A) Average swelling degree of ear (*n* = 8). (B) Average weight of granuloma (*n* = 7). (C) Pat edema volume after 1 h (*n* = 7). (D) Pat edema after volume after 4 h (*n* = 7).^######^

Mouse ear swelling models are sensitive to steroidal anti-inflammatory drugs (SAIDs) and less sensitive to NSAIDs. SA-Lip-I (1.8, 3.6 and 7.2 mg/kg) demonstrated a dose-dependent inhibition on paraxylene-induced ear swelling in mice (Figure 3(A)). The inhibition rate of the low-dose (1.8 mg/Kg) SA-Lip group has comparable therapeutic effects with the SA-I group, above all that of the middle-dose (3.6 mg/kg) and high-dose (7.2 mg/kg) group was significantly higher and significantly different from the SA-I group (*p* < .05).

#### 3.4.2. Cotton pellet granuloma in rats

Rat cotton pellet granuloma models are often used to evaluate subacute inflammation. Our results showed that SA-Lip-I effectively inhibited granulomous growth. The inhibition rate in low-dose group was similar to that in the SA-I group (25.70 and 26.57%), but significantly higher in middle- and high-dose groups (42.42 and 57.63%) (Figure 3(B)). And there were significant differences between the different groups.

#### 3.4.3. Carrageenan-induced paw edema in rats

As shown in Figure 3(C,D), the paw edema caused by carrageenan was obviously time and dose dependent. Inflammation reached a peak 2–3 h after drug administration, and slowly subsided in 4 h. The paw edema was more severe in NS group 4 h after drug administration. In SA-Lip-I low-, middle- and high-dose groups, the toe swelling was significantly suppressed, with a swelling inhibition rate of 23.89%, 52.34%, and 66.16%, respectively, at 1 h after drug administration, although the inhibition rate in the low-dose group was slightly lower than that in the SA-I group (24.00%). However, the middle and high-dose SA-Lip groups were significantly better than the SA-I group and were statistically different. The swelling inhibition rate in the three SA-Lip-I dose groups respectively reached 52.73%, 73.31%, and 88.10% at 4 h after drug administration, when paw edema also most disappeared in the SA-Lip-I high-dose group, and the inhibition rate in SA-Lip-I low-dose group was almost the same as that in the SA-I group (52.73% vs. 50.16%). And the difference between SA-Lip-I middle and high-dose group and SA-I group is significant.

### 3.5. Detection of PGE_2_, TNF-α, and IL-1β by ELISA

**Figure 4.**
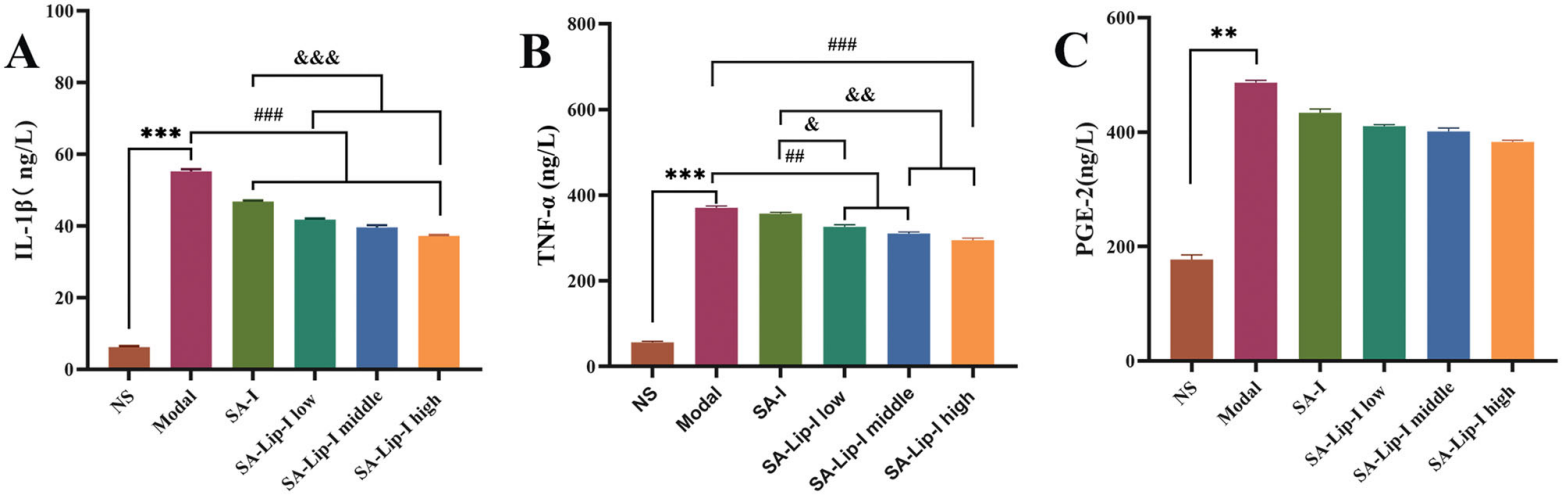
ELISA determined the PGE2, TNF-α, and IL-1β results. Compared with the control group *p < .05, **p < .01,***p < .01, compared with the model group *p* < .01, *p* < .001, compared with the SA-I group *p* < .05, *p* < .01, *p* < .001. (A) ELISA determined the IL-1β results. (B) ELISA determined the TNF-α. (C) ELISA determined the PGE-2.^#####&&&&&&^

The three inflammatory factors associated with inflammatory cells were investigated to see whether SA affected their release. The results showed (Figure 4) that carrageenan injection in the rat toe significantly increased the level of PGE2, TNF-α, and IL-1β. Compared with the model group, SA-Lip-I reduced the TNF-α and IL-1β level in a dose-dependent manner. The PGE2 level was also reduced, and more importantly, the degree of inhibition in all the three SA-Lip-I groups was higher than that in the SA-I group.

### 3.6. Safety assessment

#### 3.6.1. IV irritation evaluation

Marginal auricular testing was conducted in New Zealand big-ear rabbits to compare difference in venous irritation between NS, SA-I, and SA-Lip-I. Macroscopic observation showed no significant swelling, hyperemia, redness, and ulceration in NS and SA-Lip-I groups after IV injection. In contrast, obvious vascular swelling and RBC infiltration were the observed SA-I group.

**Figure 5.**
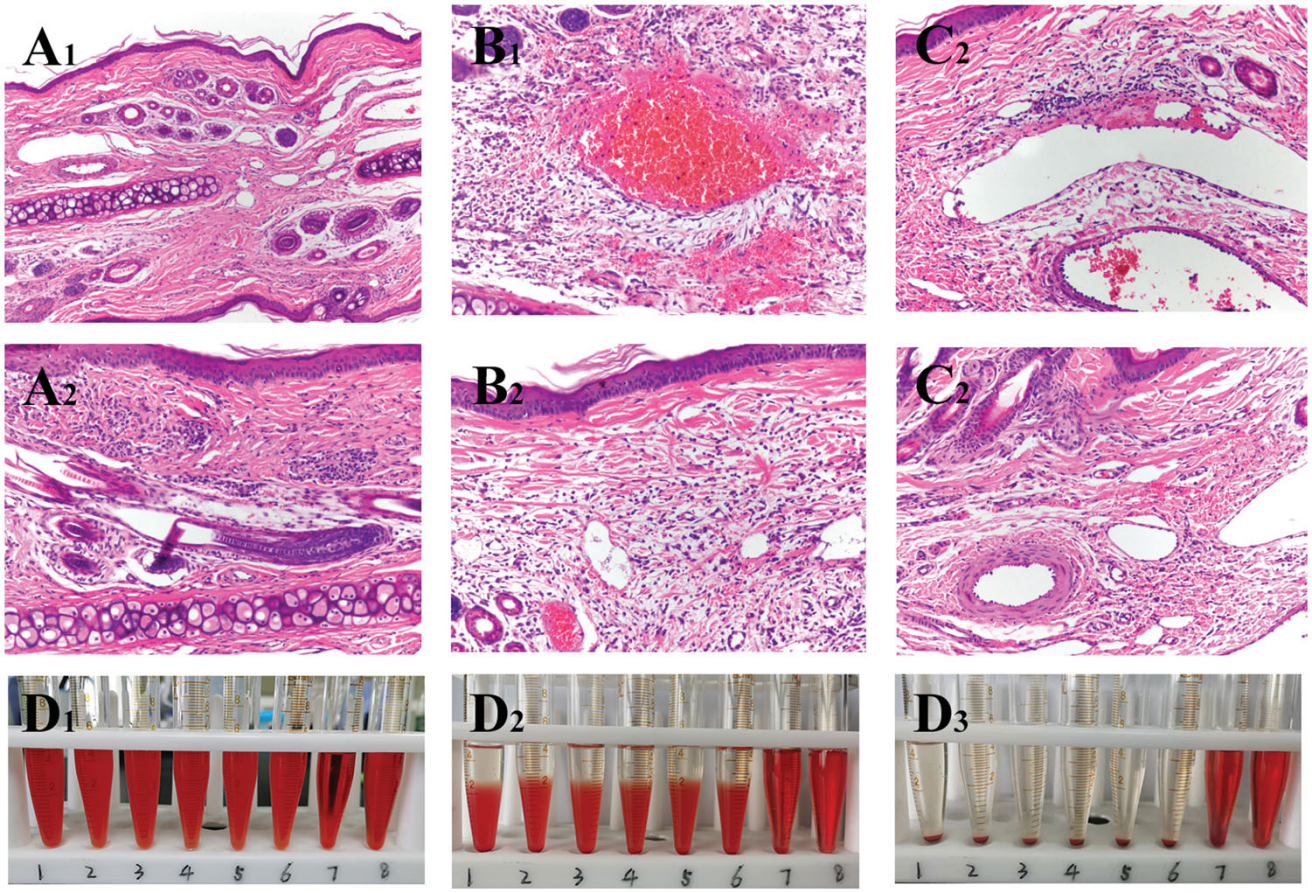
Images of rabbit pathological sections. Images of rabbit ear pathological sections. (A_1_,A_2_) NS group (200X); (B_1_,B_2_) SA-I group (200X); (C_1_,C_2_) SA-Lip-I group (200X). Hemolysis results. (D_1_–D_3_) Hemolysis results for SA-Lip-I at 0 h, 2 h and 4 h.

The results of the pathological tissue section of the injection site are shown in Figure 5(A_1_–C_3_). The rabbit ear veins in the NS group (Figure 5(A_1_,A_2_)) showed no obvious hyperemia, the vascular endothelial cell structure was intact; there was no swelling, degeneration, necrosis, or hyperplasia, there was no inflammatory change in the tube wall and surrounding tissues. The multi-layered squamous epithelium on the surface of the auricle skin showed an intact structure, and no significant lesion was observed in the deep hair follicles, sebaceous glands and sweat glands. The shape of the central elastic cartilage was normal, and no proliferation or degeneration was observed in the interstitial fibrous tissue, except for a small number of inflammatory cells.

In the SA-I group (Figure 5(B_1_,B_2_)), obvious congestion, hemorrhage, and inflammatory cell infiltration in the surrounding tissues of the tube wall were observed. In addition, venous edema, broken collagen fibers, loosening of the subcutaneous connective tissue, RBC oozing, and a large number of acute and chronic inflammatory cells were observed in the interstitial fibrous tissue of the auricle. Compared with the SA-Lip-I group and the NS control group, there are significant histopathological differences.

In the SA-Lip-I group (Figure 5(C_1_,C_2_)), there was no swelling of the endothelial cells of the ear vein, fresh blood, and a small amount of inflammatory cell infiltration were observed in the vascular wall. The fibrin of the vein wall formed grooves, and epithelial cells showed no edema, degeneration, necrosis, hyperplasia, atrophic change, or ulcer formation. No special lesion was observed in deep hair follicles, sebaceous glands and sweat gland tissues, the central elastic cartilage was morphologically normal, the chondrocytes, cartilage matrix, perichondrial structure and arteries were also normal. Histopathologically, there was significant difference between SA-Lip-I and SA-I groups.

#### 3.6.2. Hemolysis test

The results of the hemolysis experiment showed that the liquid in the positive control tube was bright red at 0 min (Figure 5(D_1_–D_3_)), and there was no RBC deposition at the bottom of T 8, indicating complete hemolysis. RBCs slowly settled at the bottom of T 6, and with time elapsing, a transparent upper layer formed, indicating that no hemolysis occurred. At 0 min, the liquid in T 7 containing commercially available SA-I looked bright red and transparent, and there was no RBC residue at the bottom of the tube, indicating that full hemolysis occurred. On the contrary, the upper layer of the SA-Lip-I in T1-5 looked clear and transparent, and RBCs settled slowly over time. After shaking, RBCs were dispersed and not hemolyzed. These results demonstrated that SA-Lip-I (0.04 mg/mL) did not cause hemolysis at 37 °C, had good blood compatibility, and could strictly prevent the occurrence of hemolytic reaction, while SA-I had a certain degree of hemolysis.

#### 3.6.3. Acute toxicity

As shown in Table 3, the toxicity of SA-Lip-I was dose dependent when the dose increased from 3.958 mg/kg to 15.01 mg/kg. Its toxicity was mainly manifested as physical action retardation and convulsions in mice. Animal death usually occurred within 3 days during the observational period, and the surviving mice recovered to a healthy state in 3 days. The estimated LD_50_ of SA-Lip-I was 9.04 mg/kg, and the 95% CI was 7.831–10.662 mg/kg versus 4.17 mg/kg and 2.17 mg/kg for SA-I as reported in the literature, indicating that SA-Lip-I is safer than SA-I.

**Table 3.** Deaths of mice in each group (*x* ± s).

Dose (mg/kg)	Number of animals	Mortality	Mortality rate (%)
3.958	10	0	0.0
4.948	10	1	10.0
6.185	10	2	20.0
7.731	10	3	30.0
9.664	10	5	50.0
12.08	10	7	70.0
15.01	10	10	100

## 4. Discussion

In this experiment, we prepared SA-Lip-I by injection-extrusion method using EPCS, ChoL and DSPE-PEG2000 as the membrane material without adding any toxic lipid film. The preparatory process is simple and the organic solvent was finally removed by ultrafiltration and freeze dried. The SA-Lip obtained showed a spherical appearance with a narrow particle size distribution (117.33 ± 0.95 nm), a PDI of 0.140 ± 0.017, a single peak and a Zeta potential of (−30.34 ± 0.23) mv, indicating that it has good dispersion performance.

SA is a water-soluble drug. When it is wrapped in the water phase of the liposome, the sealing rate will be reduced over time due to leakage of drug molecules, thus increasing irritation. Cholesterol has the characteristics to increase the rigidity of the lipid membrane (Guo et al., 2019; Szabová et al., 2021). We examined the effect of different proportions of phospholipid to cholesterol the content of organic solvents and the ratio of drug-to-lipid on liposome leakage, and finally found that three suitable mating conditions can effectively overcome this problem. This breakthrough not only well solves the problem of water-soluble drug leakage in the liposome but increases the stability of the preparation, which is more conducive to clinical use.

In this study, we selected three animal models to induce acute and chronic inflammation, including ear swelling in mice, cotton ball granuloma and cross-toe swelling in rats. In these three models, we found that the therapeutic effect was dose-dependent, and the SA-Lip-I low-dose group showed a therapeutic effect equivalent to SA-I. It is not surprising that the middle-does and high-dose SA-Lip-I. The group showed better efficacy than the SA-I group. The better curative effect of SA-Lip-I may be related to the characteristics of the liposomes. One of the most obvious characteristics of liposomes is targeting. SA enters the body in the form of liposomes, which can effectively avoid direct SA. It interacts with plasma proteins and enriches the drug in the inflammation site so that it can be swallowed by macrophages. When SA reaches the inflammation site, it can be immediately endocytosed. After fusion, the drug will be released to better exert its efficacy (Wang et al., 2009).

In this study, we mainly measured three typical inflammatory cytokines, TNF-α, IL-1β, and PGE2. They could up-regulate other inflammatory factors, promote cell proliferation and vasodilation, and play an important role in inducing and maintaining inflammation. It was found in our study that SA-Lip-I could effectively down-regulate the levels of TNF-α, IL-1β and PGE2, suggesting that SA-Lip-I may exert its anti-exudative and anti-proliferative effects by controlling release of these inflammatory factors to a certain extent. In addition, SA has no effect on immune cell function (Cheng et al., 2015; Moltu et al., 2017). In the context of *in vivo* pharmacodynamic evaluation, we consider that the efficacy of SA-Lip-I is related to the down-regulation of TNF-α, IL-1β, and PGE_2_ levels.

The safety evaluation results more intuitively indicate that SA-Lip-I is consistent with the assumption that it could effectively reduce the vascular irritation and side effects of SA. SA-Lip-I may be safer than SA-I by using the liposome as the carrier, because when SA is wrapped in the internal water phase, it can effectively avoid direct contact between the drug and the blood vessel wall, thereby reducing venous irritation. The reason for this is that SA-Lip-I is an important factor for hemolysis. SA belongs to the sodium salt of triterpene saponins, which itself has a certain degree of hemolysis, and the entrapment of liposomes can improve this problem.

## 5. Conclusion

We have successfully prepared SA-Lip-I by the simplest injection method, which has high encapsulation efficiency, small particle size and PDI, regular shape, and can maintain stability over time. In addition, SA-Lip-I has good biocompatibility with 5% glucose and 0.9% sodium chloride and can maintain stability within 24 h, which is more conducive to clinical use. Furthermore, SA-Lip-I showed significantly better efficacy and differences in the three animal models of mouse ear swelling, rat cotton ball granuloma, and rat toe carrageenan swelling as compared with SA-I. More importantly, SA-Lip-I did not induce hemolytic reaction at 37 °C, nor caused venous irritation during or after IV injection. This has a more significant advantage over SA-I. In addition, the LD_50_ of SA-Lip-I is 2.17-fold that of SA-I. Therefore, using liposomes as the carrier and encapsulating SA in its aqueous phase is a promising strategy for modifying SA, making it a safer and more effective pharmaceutical preparation for the clinical treatment of inflammation.

## Data Availability

I promise that the data in the manuscript we submit is true and reliable.
